# A Systematic Review of Cardiovascular Health Among Cancer Survivors

**DOI:** 10.3390/ijerph22060920

**Published:** 2025-06-10

**Authors:** Miriam A. Miles, Oluseun Akinyele, Abigail A. Ninson, Nicole Caviness-Ashe, Cha’Breia Means, Le’Andrea Anderson-Tolbert, Tuchondriana Smith, Reagan Coleman, Laura Q. Rogers, Joshua J. Joseph, Laura C. Pinheiro, Timiya S. Nolan

**Affiliations:** 1 Department of Medicine, Division of General Internal Medicine and Population Science, University of Alabama at Birmingham, Boshell Diabetes Building, Birmingham, AL 35233, USA; oakinyele@uabmc.edu (O.A.); chabreiameans@uabmc.edu (C.M.); leandreaanderson@uabmc.edu (L.A.-T.); colemanr@uab.edu (R.C.); lqrogers@uabmc.edu (L.Q.R.); tsnolan@uabmc.edu (T.S.N.); 2 Embrace Society, 16 Otele Avenue, East Legon, Accra 233-030, Ghana; aaninson@uab.edu; 3 Department of Engineering, Alabama State University, 915 S. Jackson Street, Montgomery, AL 36104, USA; tuchondriana2005@gmail.com; 4 Division of Endocrinology, Diabetes and Metabolism, The Ohio State University Wexner Medical Center Suite 5000 E, 700 Ackerman Road, Columbus, OH 43202, USA; joshua.joseph@osumc.edu; 5 Division of General Internal Medicine, Department of Medicine, 420 East 70th Street, Room LH-359, Box 331, New York, NY 10021, USA; lcp2003@med.cornell.edu

**Keywords:** cancer survivorship, psychosocial health, cardiovascular health

## Abstract

Cardiovascular disease (CVD) is the most common non-cancer cause of death among cancer survivors. Lifestyle and clinical factors associated with cancer mortality are also associated with cardiovascular mortality. The American Heart Association (AHA) has termed these factors “cardiovascular health” (CVH), using Life’s Simple 7 (LS7) or “Life’s Essential 8 (LE8)” to determine poor, intermediate, and high (ideal) CVH. Further, less than ideal CVH is associated with higher cancer mortality. Yet, CVH among cancer survivors remains understudied. This systematic review examined the extant literature, providing a comprehensive report of the findings addressing CVH among cancer survivors. Methods: Using PRISMA guidelines, we systematically examined CVH among cancer survivors (including patients) within PubMed, Scopus, CINAHL, and Embase databases without date limitations from June 2024 to December 2024 using the following keywords: “cancer survivors”, “cancer patient”, “cardiovascular health”, and “cardiovascular risk factors”. Two reviewers independently accessed articles in concordance with LS7 and LE8 metrics. The included studies were examined and assessed for risk of bias and synthesized to elucidate themes of CVH among cancer survivors. Results: We retrieved 2935 studies examining breast, gynecological, endometrial, prostate, colon, lung, lymphoma, and skin cancer survivors published from 2002–2024. Overall, 10 studies met criteria utilizing LS7 or LE8 CVH health outcomes (4 LS7, 5 LE8, and 1 LS7/LE8), ages 40–70 years, with a population (n = 35,980) consisting of mostly female, non-Black individuals; mean survivorship was 7.2 years. Four themes emerged: CVH outcomes among cancer survivors, social factors impacting CVH outcomes, associations of CVH, and other health outcomes opportunities for CVH awareness. Conclusions: We found that cancer survivors frequently report less than ideal CVH outcomes and would benefit from education/empowerment to support lifestyle changes that improve CVH.

## 1. Introduction

Over 18 million Americans are cancer survivors, anyone who has ever been diagnosed with cancer, no matter where they are in the course of their disease, and the number is projected to rise to about 26 million by 2040 [1,2]. Americans aged 65 years and older make up more than half of all cancer survivors [3]. Although there have been improvements in cancer screening, diagnosis, and treatment options over the past decades, cancer survivors are at a higher risk of developing cardiovascular diseases (CVD), including myocardial infarction, pulmonary embolism, stroke, and heart failure, when compared to the general population [4,5,6,7,8]. In a recent study, cancer survivors, at least 3 months after curative treatment, were found to have a 42% increased risk of developing new CVD compared to those who did not have cancer [7].

CVD leads to an increased risk of morbidity and may negatively impact the quality of life among cancer survivors compared to the general population [8,9,10]. A 2020 study showed cancer survivors with CVD were more than two times more likely to die compared to those without CVD [11,12]. The CVD complications and treatment regimens, which may cause cardiac toxicity, are associated with the increased risk of CVD-related death in cancer survivors [6,13]. Furthermore, cancer survivors, especially those who are aged 50 years and older, have an increased risk of hypertension which increases the risk of heart failure, other CVD complications, and death [14]. This study underscores the critical need to examine cardiovascular health (CVH) risks among adult cancer survivors to reduce overall CVD risk.

Cancer and CVD have shared risk factors [5,15]. Lifestyle behaviors, including diet, physical activity, sleep, and smoking, and clinical factors, including body weight, blood pressure, hemoglobin A1C, and cholesterol, are factors associated with both cancer and CVD mortality [16]. While previous oncology studies have addressed CVD and cardiovascular factors among cancer survivors, knowledge is limited about comprehensive CVH in this population. The American Heart Association (AHA) defines CVD as the presence of disease in the heart and blood vessels. However, the AHA has identified 8 measures, known as Life’s Simple 7 (LS7; blood pressure, glucose, cholesterol, body mass index, physical activity, and smoking) and Life’s Essential 8 (LE8; LS7 metrics with the addition of sleep), as metrics that can be used to conceptualize CVH [17,18]. The score derived from these measures can be used to assess an individual’s cardiovascular fitness and risk of mortality. To our knowledge, there is limited research reporting LE8 as a metric for CVH among cancer survivors. While it is well-known that ideal CVH in the general population is associated with better cardiovascular outcomes and less cardiovascular mortality [1], little is known about CVH among cancer survivors [3]. This systematic review aims to identify themes and summarize the results in extant literature related to CVH among cancer survivors.

## 2. Methods

A systematic review was conducted to provide an extensive assessment and understanding of cardiovascular health measures (LS7/LE8) among cancer survivorship among survivors utilizing The Preferred Reporting Items for Systematic reviews and Meta-Analyses (PRISMA) [19]. PRISMA is utilized in systematic reviews to specifically review systematic processes to compare and combine research findings of investigations that address the research question [19].

We searched MESH/Headings and keywords for terms associated with behaviors and factors that specifically detailed CVH as “Life’s Simple 7 (LS7)” or “Life’s Essential 8 (LE8)”, providing a metric to categorize CVH within categories (e.g., poor, intermediate, and high [ideal]). Other key terms included “cancer survivors”, “cancer patients”, and survivorship”. Given the central focus on the United States’ population and language understood by our reviewers, we limited the review to English-language literature identified in PubMed, Embase, and CINAHL databases for relevant articles from any time before December 2024. All articles were imported into Covidence Software (version 2025), for reference management [20].

Articles were screened and reviewed based on inclusion criteria to meet the study purpose [21]. Inclusion criteria covered articles that reported quantitative results for LS7 and LE8 metrics, including physical activity, diet (such as fruit and vegetable consumption), cholesterol, blood pressure, body mass index, smoking, sleep, and glycemia (glucose or hemoglobin A1c) within adult survivors of any cancer type. Interventional and non-interventional study designs were eligible for inclusion (See Table 1). These included both experimental designs, such as randomized controlled trials and cluster randomized trials, as well as quasi-experimental designs such as variations of pre- and post-tests with or without a comparison group. Articles were excluded if they did not meet inclusion criteria.

The outcomes of interest were LS7 and LE8. Data was reported for each biometric. The mean LS7 and LE8 scores were calculated to determine the relation between CVH and overall wellness among cancer patients and survivors. In addition, we applied subgroup analysis and meta-regression to explore possible causes of heterogeneity. Subgroup analysis was used to compare the treatment effects across subgroups to determine if there are significant differences, potentially indicating factors influencing the overall heterogeneity, and examine each study in subgroups based on certain characteristics (e.g., study design, patient characteristics). Meta-analyses within each subgroup were used to explore study-level data via mean age of patients, sample size, and geographical location as independent variables.

Sensitivity analyses were conducted to assess the findings and synthesize by exploring how CVH health outcomes and awareness. Results of these analyses, e.g., effects of excluding a single study at one time, are presented via the PRISMA diagram to display the stability of results. The objective was to determine whether the results of the main analysis are identical across different alternative re-analyses.

### Quality Assessment

Following identification, two reviewers (OA and TSN) performed a critical appraisal (quality assessment) of each of the included articles using the National Heart, Lung, and Blood Institute Study Quality Assessment Tools from the National Institute of Health (NIH) (See Table A1) with a modified approach. The quality assessment of each study used quantitative research criteria. Two researchers individually reviewed each article and resolved discrepancies (OA, TN).

This NIH tool evaluates internal and external validity, in addition to sources of bias, confounding, and other potential flaws specific to each study design. By affirming or negating each query in the tool, studies were assessed for an overall quality rating of “good”, “fair”, or “poor”. Our team determined “good” studies to be those affirming at least 88% (7 out of 8) of the items in the assessment tool. No studies were excluded based on the quality rating. Questions from the quality assessment tool are included in Appendix B. For data synthesis, data was extracted from selected articles and placed into matrices for content analysis of study quality, study design, study purpose, description of sample, and study findings.

## 3. Results

**Figure 1:**
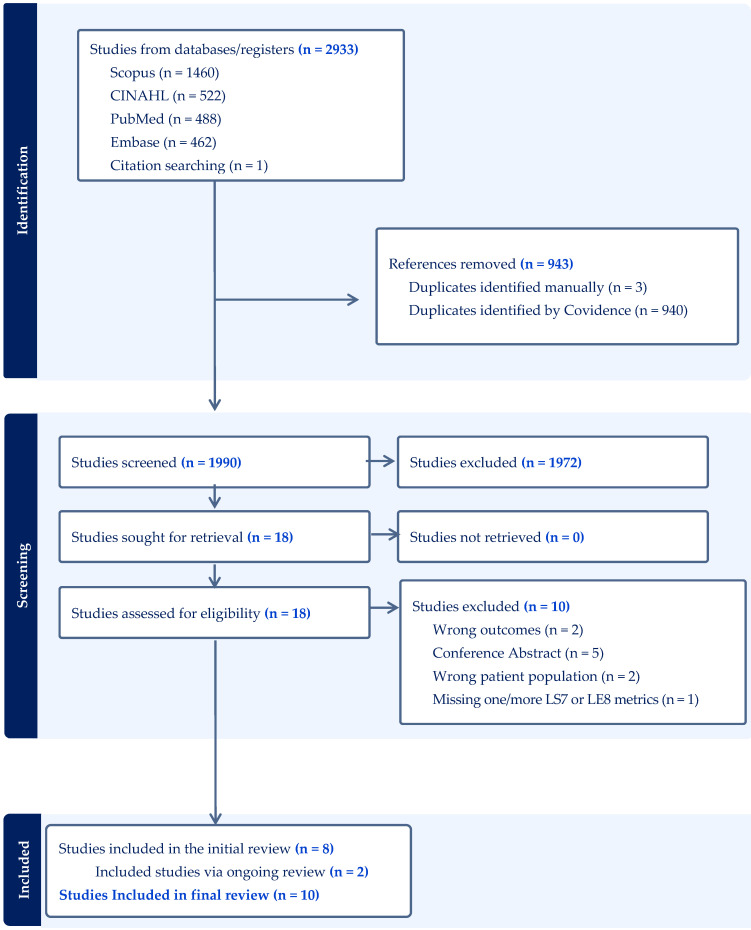
Exploring cardiovascular health outcomes among cancer survivors.

The following describes the iterative process of review. As of December 2024, a total of 2933 citations were gathered through the initial literature search in PubMed, CINAHL, and Embase. Post removal of 943 duplicate articles, two reviewers (TSN and OA) independently reviewed each of the 1990 articles’ titles and abstracts for inclusion and exclusion. Disagreements were resolved through review and consensus by all reviewers. We excluded 1972 citations from irrelevant articles based on a review of titles and abstracts. The remaining 18 articles underwent full-text review in the same manner based on inclusion and exclusion criteria. Ten additional studies were excluded based on full-text reviews, leaving ten articles that met the inclusion criteria. We then performed ascendancy and descendancy searches to identify relevant articles for inclusion. Two additional articles were identified for a total of ten included articles. The complete results of the search and review process are detailed in the PRISMA flow diagram (Figure 1). Following this analysis, 2 additional articles were added that met the inclusion criteria for a total of 10 articles analyzed at full review.

### 3.1. Setting and Study Design

This systematic review study design explores the extant literature on CVH among cancer survivors utilizing the P.I.C.O. (population, intervention, comparison, and outcomes) framework. The P.I.C.O. framework provided a basis to examine quantitative studies by accessing CVH via LS7 and LE8 scores among adult cancer survivors. To date, ten distinctive studies describe this phenomenon. The study population consisted of breast (n = 6), prostate (n = 3), endometrial/gynecologic (n = 2), colon/colorectal (n = 3), lung (n = 2), lymphoma (n = 1), skin (n = 2), and unspecified/other cancer (n = 2) survivors with a mean age range of 46–70 years from 2002–2022. Only one study substituted alcohol use to assess diet change [27].

Here we provide a detailed summary of findings on study quality, various study characteristics, and themes derived from study findings.

### 3.2. Study Population

**Figure 2:**
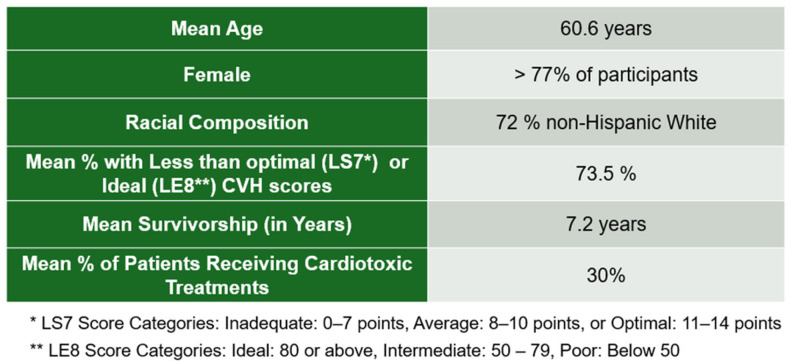
Demographics from the systematic review.

The range of mean ages reported in the studies was 40–70 years of age. The population (n = 35,980) consisted mostly of female, non-Black individuals. Mean survivorship in years is 7.2 years, with a greater number of women (77.1%). The investigation outcomes of ten studies included four LS7 (40%), five LE8 (50%), and one that assessed both LS7 and LE8 (10%). Cancer survivors reported receiving cardiotoxic treatments such as anthracycline and other cardiotoxic treatments in 80% of reviewed studies [32]. One study included both cancer survivors and oncology providers (oncologists, physician assistants, and nurse practitioners) to assess awareness of CVH risk factors among survivors [23]. Moreover, 50% of the studies reviewed only assessed women with breast cancer [23]. Over half the studies consisted of populations that were more than 70% identified as white, See Figure 2 [23,24,25,26,29,30]. Only one of the studies targeted minoritized or marginalized groups [22].

### 3.3. Setting

Data was derived from national and regional cohort studies (i.e., the National Health and Nutrition Examination Survey [NHANES], Southern Community Cohort Study [SCCS], U.S. National Health Interview Survey [NHIS], Women’s Circle of Health Follow-Up Study, the NCI Oncology Research Program, Women’s Health Initiative, and electronic health records). All participants in these studies were derived from across the U.S.

### 3.4. Review Summary

Eight out of 10 articles reported CVH outcomes among cancer survivors receiving cardiotoxic treatments. Additionally, 8 of 10 articles include breast cancer survivors within the population. Less than ideal CVH in cancer survivors ranged from 55% to 93%. Concerning the study designs, only 20% of the studies were interventional [23].

### 3.5. Themes

We identified themes by reviewing each study for recurring patterns, key concepts, and major areas of discussion across different research works. The research team reviewed the final ten articles to confer upon the patterns extracted from the analysis. Four main themes emerged from a synthesis of findings from the ten included articles: CVH outcomes among cancer survivors, social factors impacting CVH outcomes, associations of CVH and other health outcomes, and opportunities for CVH awareness among cancer survivors. A detailed exploration of each theme is described here.

### 3.6. CVH Outcomes Among Cancer Survivors

The ten articles demonstrated CVH challenges among cancer survivors, as more than 50% of cancer survivors had less than optimal (LS7 score < 10) or ideal (LE8 score < 80) CVH outcomes. Utilizing data from the NCI Community Oncology Research Program reported and found that 48.3% of cancer survivors had high cholesterol, 47.8% had hypertension or high BP, 33.1% had obesity, and 20.5% had diabetes when assessing LS7 metrics. In addition, these same metrics were assessed in patients and providers from a cancer survivor clinic and found among survivors less than ideal LE8 CVH scores in BMI, dietary practices, and physical activity categories [23,28].

Specifically, moderate CVH (mean score of 62.0 for LE8) was found within the Women’s Health Initiative cohort and other reviewed studies in general [23]. The reported mean LE8 score was less than 66 [30]. This investigation also found lower CVH scores among older participants, identified as a minority, and had lower educational attainment.LS7 and LE8 are integral in improving cancer survivorship, as higher LE8 scores before cancer diagnosis were significantly associated with a decreased risk of incident CVD in women with breast cancer, specifically.

### 3.7. Social Factors Impact CVH Outcomes

One salient theme was the impact of the social determinants of health (SDOH; e.g., age, income, education) on CVH in cancer survivors in 5 out of 10 studies. This study assessed the AHA’s LE8 framework to reveal aggregated SDOH scores across 38 domains of economic stability, neighborhood, community and social context, food poverty, education, and access to healthcare [26]. Favorable conditions were scored 0, whereas unfavorable conditions received a score of 1. Subsequently, higher aggregated SDOH scores indicated worse deprivation, which is disproportionate among young, female cancer survivors [22]. Particularly, less favorable SDOH were associated with less-than-ideal CVH (LE8 < 80, LS7 < 5). Those with a high school education more often had less than ideal CVH as compared to those with more education [26]. Most subjects were older males with higher rates of hypercholesterolemia and were less likely to report a family with low income. Younger female cancer survivors with more deprived SDOH were more likely to have suboptimal CVH [26]. However, ideal CVH was similar between Black women (33%) and White women (37%) in the SCCS; there was no significant association between race and CVH on the incidence of CVD [22].

Finally, the authors hypothesized that social disadvantage is a mediator of CVD risks among cancer survivors. Inadequate CVD risk management in cancer survivors could lead to early death among cancer survivors. Worsening social disadvantages, specifically assessing SDOH, are associated with higher cardiovascular mortality. This review found that greater social disadvantage and deprivation were independently associated with worse CVH, except for education [26]. This investigation found food insecurity to have the strongest association between social disadvantage and deprivation among cancer survivors [26].

### 3.8. Associations of CVH and Other Health Outcomes

Two papers reported on the association between CVH and cancer mortality [25,30], other CVH comorbidities, and severe psychological distress (SPD). SPD was associated with worse CVH among cancer survivors. There was also found to be an increased risk of cardiovascular death resulting from psychological stress following a new cancer diagnosis [15]. Moreover, the impact of psychological distress can lead to worse physical side effects such as lack of sleep, fatigue, a decrease in the overall quality of life, and undue delay in cancer treatments and other medical care [33].

An independent review of LE8 scores demonstrated that low-CVH vs. high-CVH groups had the largest association with a 55% increased risk of myocardial infarction and coronary heart disease among breast cancer patients [30]. Similarly, this association was disproportionately stronger (1.57, *P interaction* = 0.026) in younger females (ages 18–45 years) compared to older females (ages 46 and older) [11]. There were dependent associations between the highest quartile of the SDOH score and all components of CVH, with the strongest association observed for inappropriate sleep duration [26]. The association between CVH and SPD remained significant among different cancer types (breast, prostate, colon/rectum, and melanoma). An NHANES study of LE8 metrics revealed that individuals with lower CVH scores (e.g., LE8 < 60) had significantly higher rates of all-cause, cancer-specific, and non-cancer mortality compared to those with higher CVH scores (LE8 > 60) [28]. Individuals with better CVH had lower chances of dying from cancer or other non-cancer-related conditions. Specifically, the accumulated risk of death at follow-up was significantly lower in the high CVH group [25]. This study reported a risk reduction of death from all causes associated with a higher LE8 score within the range between 61.25 (HR: 0.76, 95% CI, 0.59–0.98) and 100 points (HR: 0.28, 95% CI, 0.12–0.62) [25]. Furthermore, older survivors were found to be at increased risk for mortality compared to younger survivors at the same CVH level [31].

### 3.9. Opportunities for CVH Awareness Among Cancer Survivors

The fourth theme was the lack of CVH awareness among individuals living with cancer survivors. While CVH is not likely to be ideal among cancer survivors (less than 50% had ideal LS7 or LE8 scores), and there are known social factors that influence CVH, cancer survivors report having or receiving little CVH awareness after their cancer diagnosis [23]. This review demonstrated that CVH awareness among cancer survivors (and cancer health care providers) was not pervasive: more than 60% of cancer survivors were not aware of CVH risk factors [23]. Further, only 50% of healthcare providers reported discussing CVH with post-treatment patients [23]. Additionally, female, younger cancer survivors were more likely to have less than ideal CVH scores and reported being unaware of CVH factors [22,32]. This work found an increased prevalence of multiple CV comorbidities among cancer survivors who also reported an interest in discussing CVH awareness with providers [22,32]. Moreover, a limited number of interventions were identified aimed at addressing CVH awareness among cancer survivors.

## 4. Discussion

Cancer survivors have increased CVH risk due to iatrogenic treatments. Moreover, cancer survivors require a different approach to CVH, as this is closely linked with comorbidities such as diabetes, hypertension, and obesity. These conditions often coexist and exacerbate each other, leading to worse outcomes for individuals with cardiovascular disease (CVD). Specifically, an increased burden of cardiometabolic comorbidities is associated with increased risks of hospitalization and adverse outcomes in the general population. Addressing these comorbidities is crucial for improving CVH [e.g., blood pressure control, cholesterol management, and smoking cessation], and other strategies involve promoting physical activity, healthy diets, and access to preventive care. Community-based actions also address disparities by targeting underserved populations and improving access to healthcare.

LS7 and LE8 metrics provided a framework to assess CVH outcomes among cancer survivors. However, this systematic review revealed that the assessment of LS7 and LE8 metrics among cancer survivors has not been well utilized to date. Scant findings indicated that cancer survivors are likely to report less than ideal CVH, face social factors associated with CVH, report knowledge gaps around CVH after cancer, and report comorbid conditions. Thus, there is an urgent need to assess and address CVH among cancer survivors, as CVH risks were higher compared to individuals without a cancer diagnosis [5]. Decreased CVH awareness was associated with increased CVH risks among cancer survivors.

It is well known that cancer and its treatment can lead to the development of CVD [5]. For example, previous investigations found that women with breast cancer, especially those who underwent cardiotoxic treatment, may face elevated CVH risks, indicating a greater need to assess the inherent behaviors, medical, socioeconomic, or cancer treatment exposures that may contribute to increased risk of CVD [30]. As CVH is associated with the development of CVD, understanding CVH in cancer survivors may be beneficial to avert later development of CVD. Unfortunately, we found that the extant literature reported that CVH among cancer survivors (much like the general population) was less than ideal [32] Studies examined in this review predominantly included breast cancer survivors, yet there are over 200 types of cancer with a myriad of treatment options [30]. Furthermore, less than CVH was associated with poorer outcomes for cancer survivors. More research is needed to inform the knowledge base around the intricacies of cancer, cancer types, cancer treatment, and CVH.

Regarding CVH outcomes among cancer survivors, this population faces distinctive challenges in maintaining optimal CVH. Cancer treatments, such as chemotherapy and radiation, may have cardiotoxic effects, increasing the risk of poor CVH. These risk factors may further contribute to less optimal LS7/LE8 metrics among cancer survivors.

Social determinants of health encompass the economic, social, psychosocial, and environmental factors that affect the health conditions into which people are born and live that affect their health [34]. Previous studies have shown that SDOH influences the development of cardiovascular risk factors and outcomes [35,36,37]. As CVD accounts for 35% of female deaths annually [38], it is imperative to address underdiagnosed and undertreated CVD due to misconceptions and lack of awareness. Social determinants of health, such as socioeconomic status, education, and access to healthcare, significantly impact the health outcomes of cancer survivors [39]. These factors can lead to disparities in cancer care, with marginalized groups experiencing higher cancer incidence and mortality rates [39]. Creating novel targeted interventions is crucial for improving health equity and outcomes for Black women [39].

Our study revealed that age, SES, and education were linked to CVH among cancer survivors. Previous studies have seen an inverse relationship between CVH risk factors and age [31,40]. Furthermore, our study revealed that younger female cancer survivors with lower SES had suboptimal CVH. Given the limited data, there is little ability to investigate the interplay between age and SES among cancer survivors. There is also a. need for additional investigation of potential factors associated with social determinants of health tied to the construct of race.

In addition, the SDOH factors in the study revealed associations between CVH and other health outcomes. Significant associations have been found between mortality, development of comorbidities, and SPD. Intentionally addressing cardiovascular disease (CVD) and other comorbidities is fundamental to improving cancer survivors’ health outcomes and enhancing quality of life. The AHA emphasizes the importance of addressing psychosocial stress (including depression and anxiety) as a factor that increases the risk of developing CVD, highlighting the need for comprehensive care that includes mental health management.

This study identified a gap in CVH awareness among cancer survivors to address CVH and associated outcomes among cancer survivors. Generally, poor health literacy is associated with poorer health outcomes [41]. With the growing number of cancer survivors, low CVH awareness may become a public health concern [41]. This review identified specific gaps regarding survivors’ understanding of their risk of heart disease and the lack of knowledge of one or more of their cardiovascular health risk factors. Among these gaps were underestimation of CVH risks, the lack of communication by healthcare providers regarding cancer survivors’ risks, inadequate follow-up care of CVH risk factors, and health literacy. Particularly, interventions have included community health workers to address health literacy challenges in CVD, with successful improvement in outcomes [42]. Moreover, there is a need for enhanced awareness of cardiovascular health among cancer survivors and oncology providers. Cancer survivors would benefit from post-treatment CVH assessments and education on CVH risks. This same work can be promising when applied to cardiovascular health among cancer survivors.

Addressing CVH among cancer survivors represents an opportunity to improve CVH metrics among cancer survivors. To date, cardio-oncology is an emerging field with very few guidelines for the comprehensive management of CVH aside from those treated with anthracyclines. Given the complex and multifaceted nature of CVH among cancer survivors, a multi-level approach including CVH education, routine CVH assessment, and interventions is needed. This review revealed that often this overlooked population would greatly benefit from community-level interventions aimed at producing optimal cardiovascular health outcomes for cancer survivors, as none of the articles reviewed were community-based [30].

Similarly, in non-cancer studies, community-engaged lifestyle interventions addressing social needs have shown promise in improving CVH. Cardiovascular health (CVH) interventions are crucial for cancer survivors, who face nearly twice the risk of fatal heart disease compared to the general population. The 24-week Black Impact study was found to increase LS7 CVH scores by 0.67 and 0.93 for weeks 12 and 24, respectively [43]. Effective translation of these interventions into routine care is essential, as many survivors have multiple cardiovascular risk factors, such as hypertension, obesity, and diabetes. Integrating CVH assessments and counseling into oncology follow-up can significantly improve survivors’ long-term health outcomes. Tools such as the American Heart Association’s LS7 and LE8 can be adapted for cancer survivors to facilitate discussions and ensure cancer survivors receive comprehensive care that addresses both their cancer and cardiovascular health needs [32].

Future directions should assess strategies to educate both cancer survivors and healthcare providers. It is important to identify the cardiovascular health risks of cancer survivors and determine the most effective manner to promote CVH-related health education and awareness, along with proper communication from providers. Future research should develop and test targeted health education materials among cancer survivor populations and providers. These interventions should be incorporated alongside additional strategies to decrease CVH-related cancer disparities. Community- and peer-based interventions are a novel and engaging approach to decreasing racial and ethnic disparities, increasing CVH awareness, and addressing other health outcomes among cancer survivors.

The AHA’s dedication to cardio-oncology rehabilitation aims to address cardio-oncology equity in cancer care and research by examining cancer patients and survivors at high risk for poor CVH [44]. Moreover, interventions are needed to address the unique exposures and complications related to cancer care. Furthermore, this emphasizes the need to educate and empower cancer survivors and healthcare providers to make modifiable changes to improve CVH and CVH awareness.

## 5. Limitations

The findings from this review must be carefully considered within the context of several limitations. First, there were only ten articles identified that met the inclusion criteria. These articles described CVH but had varying sample characteristics that limit the overall assessment of CVH. Another limitation was the lack of racial diversity, as many included mostly white, female participants. Additionally, only quantitative studies were analyzed in this review. Hence, this investigation only included studies conducted in the United States and so does not present an international assessment of CVH risks among cancer survivors.

The most common limitations in quality were the cross-sectional study designs, limited sample size (e.g., n = 49, n = 345), and possible recall bias of study participants. Three of the studies reported that the sample size was not adequate to detect outcome differences with sufficient power [22,23]. In addition, reclassification of the dietary variable was necessary for the Chan et al. articles to calculate the LE8 score. Notably, compared to pre- and post-tests and other study designs, randomized trials were more likely to have an overall quality rating of good to fair. Lastly, there is a lack of data regarding specific cancer treatment agents and/or precise targets for radiation therapy, along with other considerations that may impact CVH among cancer survivors [25,29,30].

## 6. Conclusions

Our review found that cancer survivors may have an increased risk of less-than-ideal CVH outcomes. This is important given the comorbid and iatrogenic nature of cardiovascular disease among cancer survivors. Future research requires greater engagement of underserved and underrepresented participants. High-quality, generalizable studies are of vital importance in identifying strategies to achieve health equity in CVH among cancer survivors. Likewise, this systematic review highlights opportunities for academic-community collaborations in addressing disparities among cancer survivors, gaps in CVH awareness and community-based participatory research, and various interventions addressing health outcomes.

As a result, cancer survivors often require tailored approaches to CVH management. CVH awareness among cancer survivors, including routine cardiovascular risk assessments during oncology follow-ups, is key to addressing CVH among cancer survivors. Additionally, incorporating CVH discussions into cancer care can bridge gaps in knowledge and improve CVH outcomes among this population.

Lastly, addressing structural and social determinants of health barriers such as lower income and health education levels may limit access to care. Geographic challenges, especially in rural areas, CVH awareness, and financial constraints may also impact access to specialized cardio-oncology education and services. Additionally, structural issues such as limited healthcare provider awareness of cardio-oncology needs may delay care in addressing CVH among cancer survivors.

This review provided much-needed evidence to support future research addressing social determinants of health, CVH awareness, and associations with psychological distress and other health outcomes. Yet, based on the available studies, there is insufficient data to recommend a specific community-engaged or CBPR intervention to improve the attainment of AHA LS7 and LE8 metrics among non-white males and females. Future community-based interventions measuring AHA’s LS7 and LE8 metrics to reduce disparities in CVH risk and address social determinants of health impacting hypertension, diabetes, and mortality rates among cancer survivors.

## Figures and Tables

**Table 1:** Summary of the articles’ description, data collection, cancer diagnosis, and primary outcomes results.

Author (Year)	Purpose	*N*	Databases and Dates of Data Collection	Cancer Diagnosis	Study Design	Primary Outcome Results
Nolan, T., et al. (2024) [22]	To examine if racial differences in cardiovascular health (CVH) are associated with CVD disparities among women with breast cancer and gynecologic cancers.	345	Southern Community Cohort Study (SCCS), 2002–2009	Breast and Gynecological	Cross-sectional analysis	Ideal CVH was similar between Black women (33%) and White women (37%). Race and CVD were not associated with CVD incidence.
Weaver K., et al. (2021) [23]	To evaluate survivors’ awareness of cardiovascular risk factors and examine the usability of a novel electronic health record-enabled cardiovascular health tool from the perspective of both breast cancer survivors and oncology providers	69 (49 breast cancer survivors; 20 oncology providers)	Participants were recruited from the survivorship clinic	Breast	Interventional, Mixed-methods design	Over 90% of breast cancer survivors thought the tool improved their understanding of CVH risk. More than 85% of oncology providers agreed it would help their effectiveness and said they would use the tool most/all of the time when providing survivorship care.
Weaver K., et al. (2024) [24]	To assess survivor CVH profiles, compare self-reported and electronic health record-based categorization of CVH factors and describe perceptions regarding addressing CVH during oncology encounters	502	NCI Community Oncology Research Program 2020–2022	Breast, Prostate, Colon/Colorectal, Lymphoma, and Endometrial/ Gynecologic	Interventional Cross-sectional analysis	Most participants had breast cancer (79.7%). 48.3% had high cholesterol, 47.8% had hypertension or high BP, 33.1% had obesity, and 20.5% had diabetes. 30.5% of survivors received high cardiotoxicity potential cancer treatment. 48% had ideal levels of PA, BMI (18.9%), cholesterol (17.9%), BP (14.1%), healthy diet (11.0%), and glucose/hba1c (6.0%).
López-Bueno, R., et al. (2024) [25]	To examine the dose-response association between cardiovascular health (CVH) and risk of all-cause, cardiovascular disease (CVD), and cancer mortality among cancer survivors	1701	National Health and Nutrition Examination Survey (NHANES), 2007–2018	Not specified	Prospective cohort study	Dose-response association between LE8 score and all-cause mortality with significant risk reductions within the range between 61.25 (hazard ratio [HR]: 0.76, 95% CI, 0.59–0.98) and 100 points (HR: 0.28, 95% CI, 0.12–0.62). No significant dose-response association was observed between LE8 and cancer deaths.
Satti, D., et al. (2024) [26]	To investigate associations between the social determinants of health (SDOH) and CVH of adult cancer survivors	8254	National Health Interview Survey (NHIS), 2013–2017	Breast, Prostate, Colon/Colorectal, Lung, Skin, and Not specified	Cross-sectional	Worse SDOH was associated with worse CVH (risk ratio 1.30; 95% CI: 1.25–1.35; *p* < 0.001).
Chan, S.K., et al. (2023) [27]	To investigate the relationship between psychological distress and cardiovascular health among cancer survivors	11,932	National Health Interview Survey (NHIS), 2013–2017	Unknown/Not specified	Cross-sectional analysis	Severe SPD was independently associated with worse cardiovascular health [adjusted RR 1.24 (1.19–1.29), *p* < 0.001].
Fan, C., et al. (2024) [28]	To investigate Life’s Essential 8, a measure of cardiovascular health, associations with mortality outcomes in cancer survivors	1818	National Health and Nutrition Examination Survey (NHANES), 2005–2018	Unknown/Not specified	Retrospective cohort	Individuals with high CVH had significantly lower hazard ratios for all-cause, cancer-specific, and non-cancer mortality compared to those with low CVH, with HRs of 0.42 (0.35, 0.51), 0.52 (0.35, 0.77), and 0.37 (0.30, 0.47), respectively. Significant interactions between LE8 and Poverty Index Ratio (PIR) for probability of cancer-specific and non-cancer-specific death (*p* < 0.05)
Guo A., et al. (2020) [29]	To investigate the receipt of radiation alongside other types of cancer therapies on the risk of CHD and mortality using novel statistical techniques	1934	Electronic Health Records, 2006–2007	Breast	Retrospective cohort	The joint effect of poor CVH and receipt of cardiotoxic treatments on CHD (75.9%) and death (39.5%) was significantly higher than their independent effects [poor CVH (55.9%) and cardiotoxic treatments (43.6%) for CHD, and poor CVH (29.4%) and cardiotoxic treatments (35.8%) for death].
Wadden, K., et al. (2024) [30]	To evaluate the incidence of CVD in relation to the Life’s Essential 8 (LE8) score among women with BC	7165	Women’s Health Initiative (WHI), 2005–2025	Breast	Cross-sectional, Retrospective cohort	The risk of CVD events was highest for low CVH compared with moderate and high CVH.
Liu, W., et al. (2024) [31]	To investigate the association between LE8 and the prognosis of cancer survivors	2191	National Health and Nutrition Examination Survey (NHANES), 2005–2018	Breast, Prostate, and Not Specified	Cross-sectional, Retrospective cohort	A higher LE8 score was independently associated with a decreased risk of both all-cause and cardiovascular mortality in cancer survivors.

## Data Availability

No new data were created or analyzed in this study. Data sharing is not applicable to this article.

## Appendix A

**Table A1:** Questions and Guidance for NIH-NHLBI Study Quality Assessment Tool.

Criteria	Yes	No	Other (CD, NR, NA) *
1. Is the review based on a focused question that is adequately formulated and described?			
2. Were eligibility criteria for included and excluded studies predefined and specified?			
3. Did the literature search strategy use a comprehensive, systematic approach?			
4. Were titles, abstracts, and full-text articles dually and independently reviewed for inclusion and exclusion to minimize bias?			
5. Was the quality of each included study rated independently by two or more reviewers using a standard method to appraise its internal validity?			
6. Were the included studies listed along with important characteristics and results of each study?			
7. Was publication bias assessed?			
8. Was heterogeneity assessed? (This question applies only to meta-analyses.)			
**Quality Rating (Good, Fair, or Poor)**			
Rater #1 initials:			
Rater #2 initials:			
Additional Comments (If poor, please state why):			

* CD, cannot determine; NA, not applicable; NR, not reported.

## Appendix B

### Guidance for Quality Assessment Tool for Systematic Reviews and Meta-Analyses

A systematic review is a study that attempts to answer a question by synthesizing the results of primary studies while using strategies to limit bias and random error. These strategies include a comprehensive search of all potentially relevant articles and the use of explicit, reproducible criteria in the selection of articles included in the review. Research designs and study characteristics are appraised, data are synthesized, and results are interpreted using a predefined systematic approach that adheres to evidence-based methodological principles.

Systematic reviews can be qualitative or quantitative. A qualitative systematic review summarizes the results of the primary studies but does not combine the results statistically. A quantitative systematic review, or meta-analysis, is a type of systematic review that employs statistical techniques to combine the results of the different studies into a single pooled estimate of effect, often given as an odds ratio. The guidance information below is organized by question number from the tool for quality assessment of systematic reviews and meta-analyses [45].

**Guidance for Quality Assessment Tool for Systematic Reviews and Meta-Analyses**
Question 1. Focused question
The review should be based on a question that is clearly stated and well-formulated. An example would be a question that uses the PICO (population, intervention, comparator, outcome) format, with all components clearly described.
Question 2. Eligibility criteria
The eligibility criteria used to determine whether studies were included or excluded should be clearly specified and predefined. It should be clear to the reader why studies were included or excluded.
Question 3. Literature search
The search strategy should employ a comprehensive, systematic approach to capture all of the evidence possible that pertains to the question of interest. At a minimum, a comprehensive review has the following attributes:
- Electronic searches were conducted using multiple scientific literature databases, such as MEDLINE, EMBASE, CINAHL, and Scopus
- Manual searches of references found in articles and textbooks should supplement electronic searches.
Additional search strategies that may be used to improve the yield include the following:
- Studies published in other countries.
- Studies published in languages other than English.
- Identification by experts in the field of studies and articles that may have been missed.
- Search of grey literature, including technical reports and other papers from government agencies or scientific groups or committees; presentations and posters from scientific meetings, conference proceedings, unpublished manuscripts, and others. Searching the grey literature is important (whenever feasible) because sometimes only positive studies with significant findings are published in the peer-reviewed literature, which can bias the results of a review.
In their reviews, researchers described the literature search strategy clearly and ascertained it could be reproducible by others with similar results.
Question 4. Dual review for determining which studies to include and exclude.
Titles, abstracts, and full-text articles (when indicated) should be reviewed by two independent reviewers to determine which studies to include and exclude in the review. Reviewers resolved disagreements through discussion and consensus or with third parties. They clearly stated the review process, including methods for settling disagreements.
Question 5. Quality appraisal for internal validity
Each included study should be appraised for internal validity (study quality assessment) using a standardized approach for rating the quality of the individual studies. Ideally, this should be done by at least two independent reviewers who appraise each study for internal validity. However, there is not one commonly accepted, standardized tool for rating the quality of studies. So, in the research papers, reviewers looked for an assessment of the quality of each study and a clear description of the process used.
Question 6. List and describe included studies.
All included studies were listed in the review, along with descriptions of their key characteristics. This was presented either in narrative or table format.
Question 7. Publication bias
Publication bias is a term used when studies with positive results have a higher likelihood of being published, being published rapidly, being published in higher impact journals, being published in English, being published more than once, or being cited by others. Publication bias can be linked to favorable or unfavorable treatment of research findings due to investigators, editors, industry, commercial interests, or peer reviewers. To minimize the potential for publication bias, researchers can conduct a comprehensive literature search that includes the strategies discussed in Question 3.
A funnel plot—a scatter plot of component studies in a meta-analysis—is a commonly used graphical method for detecting publication bias. If there is no significant publication bias, the graph looks like a symmetrical inverted funnel.
Reviewers assessed and clearly described the likelihood of publication bias.
Question 8. Heterogeneity
Heterogeneity is used to describe important differences in studies included in a meta-analysis that may make it inappropriate to combine the studies. Heterogeneity can be clinical (e.g., important differences between study participants, baseline disease severity, and interventions), methodological (e.g., important differences in the design and conduct of the study), or statistical (e.g., important differences in the quantitative results or reported effects).
Researchers usually assess clinical or methodological heterogeneity qualitatively by determining whether it makes sense to combine studies. For example:
- Should a study evaluating the effects of an intervention on CVD risk that involves elderly male smokers with hypertension be combined with a study that involves healthy adults ages 18 to 40? (Clinical Heterogeneity)
- Should a study that uses a randomized controlled trial (RCT) design be combined with a study that uses a case-control study design? (Methodological Heterogeneity)
Statistical heterogeneity describes the degree of variation in the effect estimates from a set of studies; it is assessed quantitatively. The two most common methods used to assess statistical heterogeneity are the Q test (also known as the X2 or chi-square test) or the I2 test.
Reviewers examined studies to determine if an assessment for heterogeneity was conducted and clearly described. If the studies are found to be heterogeneous, the investigators should explore and explain the causes of the heterogeneity and determine what influence, if any, the study differences had on overall study results.
